# Effect of Low‐Intensity Electrical Stimulation on External Apical Root Resorption and Periodontal Indices Following En‐Masse Retraction of Upper Anterior Teeth in Young Adults: A Randomized Controlled Trial

**DOI:** 10.1002/cre2.70188

**Published:** 2025-08-05

**Authors:** Rashad I. Shaadouh, Mohammad Y. Hajeer, Mohammad Khursheed Alam, Samer T. Jaber

**Affiliations:** ^1^ Department of Orthodontics, Faculty of Dentistry University of Damascus Damascus Syria; ^2^ Orthodontic Division, Department of Preventive Dentistry, College of Dentistry Jouf University Sakaka Saudi Arabia; ^3^ Department of Orthodontics, Faculty of Dentistry Al‐Wataniya Private University Hama Syria

**Keywords:** electrical stimulation, en‐masse retraction, periodontal indices, root resorption

## Abstract

**Objective:**

To evaluate external apical root resorption (EARR) and periodontal indices during en‐masse retraction of maxillary anterior teeth stimulated with low‐intensity electrical currents.

**Trial Design:**

A two‐arm randomized controlled trial.

**Methods:**

Young adult patients who visited the Department of Orthodontics at Damascus University between November 2023 and March 2024 and met predefined inclusion criteria were randomly allocated into two groups using block randomization. The study included 34 patients, with 17 assigned to the electrically stimulated en‐masse retraction (ESER) group and 17 to the conventional en‐masse retraction (CER) group. The force for the en‐masse retraction technique in both groups was 250 g per side. The force was applied through bilateral closed‐coil nickel‐titanium springs anchored to an orthodontic mini‐screw on each side. In the ESER group, each upper anterior tooth was subjected to a continuous electrical stimulation of 15–20 µA for 5 h daily, utilizing an intraoral removable electrical stimulation device. EARR was assessed using digital panoramic radiographs. Four periodontal indices were also used to assess periodontal status. Blinding was confined to data analysis.

**Results:**

Thirty‐four patients (26 females and 8 males) were analyzed with a mean age of 21.12 ± 2.41 years. At the end of the en‐masse retraction phase, no significant difference in EARR was observed between the two groups (*p* > 0.05). The observed root resorption in the ESER and CER ranged between 0.27 and 0.64 and 0.32 and 0.71 mm, respectively. Also, insignificant differences were found in all periodontal indices studied at all measurement points between the two groups (*p* > 0.05).

**Conclusions:**

Low‐intensity electrical stimulation had no significant effect on root resorption during the en‐masse retraction of the six upper anterior teeth. Both groups exhibited comparable slight root resorption without any significant difference between them. Additionally, low‐intensity electrical stimulation did not affect the periodontal status during en‐masse retraction.

**Trail Registration:**

Clinical Trials database (NCT06873490).

## 1 Introduction

Class II division I malocclusion is one of the most prevalent types of malocclusions worldwide (Alhammadi et al. 2018). It is often addressed in adult patients through camouflage treatment, which involves extracting the upper first premolars and retracting the upper anterior teeth.

Various techniques have been developed to optimize anterior retraction while ensuring effective anchorage control (Chae 2006; Hong et al. 2005; Park and Kwon 2004). The retraction of anterior teeth in Class II cases presents unique biomechanical challenges, particularly concerning anchorage control (Kuroda et al. 2009). Traditionally, intraoral appliances such as transpalatal arches and headgear have been used to reinforce posterior anchorage. Temporary anchorage devices (TADs) have emerged as a superior alternative, providing absolute anchorage and preventing unwanted mesial movement of posterior teeth (Al‐sibaie and Hajeer 2014). Skeletal anchorage provides an effective alternative method for Class II malocclusion treatment, such as maxillary distalization in adult patients. This method may overcome challenges related to patient cooperation or other potential risks such as external apical root resorption (EARR) (Ahuja et al. 2024).

The extraction space closure phase is the longest phase during the camouflage treatment. It usually takes a long time, ranging from 9 to 12 months, when using the en‐masse retraction technique (Khlef et al. 2018). Although some studies have shown the superiority of the en‐masse retraction technique over the traditional two‐stage technique due to several practical advantages (Khlef et al. 2018), it still takes a relatively long time. This long treatment time will be associated with several side effects, especially an increased risk of root resorption, periodontal diseases, and caries rate (Talic 2011).

Orthodontists continually explore methods to speed up tooth movement to minimize the overall treatment duration. Various approaches have been devised and refined, including surgical interventions that enhance the biological response of the periodontal ligament, such as corticotomy (Al‐Naoum et al. 2014), laser‐assisted flapless corticotomy (Shaadouh et al. 2022), piezocision (Alfawal et al. 2018), and periodontally accelerated osteogenic orthodontics (PAOO) (Alsino et al. 2022), as well as physical and mechanical techniques that stimulate cellular activity and expedite tooth movement, such as low‐level laser therapy (LLLT) (Hasan et al. 2022), resonance vibration (Leethanakul et al. 2016), and pulsed electromagnetic fields (Bhad Patil and Karemore 2022).

Recently, in a randomized controlled clinical trial, Shaadouh et al. showed that applying microampere electrical stimulation (15–20 Aµ/tooth) to the maxillary anterior teeth during en‐masse retraction of the maxillary anterior teeth accelerated the rate of tooth movement by 28%, which was statistically significant compared to the control group (Shaadouh et al. 2024a, 2024b). Also, in another report, Shaadouh et al. found that this acceleration technique was associated with acceptable levels of pain and discomfort and high levels of acceptance by the patients (Shaadouh et al. 2024a, 2024b). It is important to note that these articles focused on the rate of orthodontic tooth movement and patient‐centered outcomes, respectively. However, other complications that may occur during electrically stimulated en‐masse retraction (ESER) that affect the dental tissues, such as external root resorption or periodontal tissue, were not evaluated.

Root resorption and periodontal problems are some of the most frequent complications encountered during fixed orthodontic treatment (Ahmed et al. 2022; Yassir et al. 2021). EARR is a pathophysiological condition manifested by a decrease in the length of the tooth root resulting from loss of cementum and/or root dentin (American Association of Endodontists 1994). Root resorption is one of the most common complications of orthodontic treatment that may be difficult for the clinician to avoid due to its influence by many factors, such as the amount of force, the amount of tooth movement, and the duration of treatment (Ng et al. 2018; Weltman et al. 2010). External root resorption usually takes 3–4 months to become detectable by X‐ray (Levander 1998). Although it starts within 2–5 weeks of the start of orthodontic treatment (Kurol et al. 1996). It is crucial for orthodontists to identify risk factors for root resorption and develop methods to prevent such complications, especially since damage to the root surface is largely irreversible once it reaches the dentin (Lopatiene and Dumbravaite 2008).

Previously, in a controlled clinical trial, Huang et al reported root resorption during conventional en‐masse retraction (CER) of approximately 0.43 and 0.58 mm in the central and lateral incisors, respectively. Khlef et al assessed external root resorption in the roots of anterior teeth during accelerated en‐masse retraction, using either conventional or flapless corticotomy (Khlef et al. 2020). They reported that minor root resorption was observed in a small number of teeth (9.2% and 5%, respectively) after the en‐masse retraction (Khlef et al. 2020). Additionally, Yousry et al in a randomized controlled trial (RCT), examined external root resorption in cases of accelerated en‐masse retraction using LLLT. They found root resorption in all maxillary anterior teeth after the en‐masse retraction, with no significant difference in the amount of resorption between the LLLT group (1.16 mm) and the control group (1.31 mm). Similarly, Nasser et al reported comparable results about the effect of low‐level laser on root resorption during maxillary incisor intrusion (Nasser et al. 2023). It is noteworthy to know that no previous study has evaluated the effect of low‐intensity electrical stimulation on external root resorption in either en‐masse retraction or other orthodontic tooth movements.

On the other hand, fixed orthodontic devices facilitate the accumulation of bacterial plaque around their components (Alexander 1991), which releases harmful substances responsible for the majority of dental and periodontal issues (Wade 2013). Periodontal health plays a crucial role in orthodontic treatment outcomes, influencing both the stability of tooth movement and overall oral health (Kwon et al. 2024). Evaluating periodontal status is essential to identify potential risks, such as inflammation, attachment loss, and gingival recession, which may arise due to mechanical forces exerted during tooth movement. Furthermore, the relationship between unmet treatment needs and oral health‐related quality of life has been well‐documented, highlighting the necessity of comprehensive periodontal evaluation in orthodontic research (Shetty et al. 2024).

No previous study has evaluated periodontal indices with en‐masse retraction of the upper six anterior teeth enhanced by low‐intensity electrical stimulation. In contrast, Lee et al evaluated the effect of electrical stimulation on periodontal tissues using a special electric toothbrush (Proxywave, Tromatz basic, proxyhealthcare, Ulsan, Korea) during the leveling and alignment phase (Lee and Ha 2021). They found that electrical stimulation using an electric toothbrush significantly reduced the gingival index (GI) compared with a conventional electric toothbrush.

En‐masse retraction of the anterior teeth induces significant biomechanical changes that can affect both hard and soft tissues. One of the most concerning side effects is EARR, as a consequence of excessive force application or prolonged tooth movement, leading to irreversible shortening of the dental roots (Ahuja et al. 2025). Therefore, it is essential to study root resorption during mass reduction and to investigate appropriate methods to avoid these side effects. Additionally, anterior tooth inclination is notably influenced by retraction mechanics. Many studies have shown that maxillary incisors become palatally inclined at the end of en‐mass retraction (Al‐sibaie and Hajeer 2014; Khlef et al. 2020; Tunçer et al. 2017; Upadhyay et al. 2008). However, there is some discrepancy between these studies regarding the extent of palatal inclination reported. Excessive tipping rather than bodily movement can alter the force distribution along the roots, potentially exacerbating resorptive processes. Beyond these dental changes, retraction‐related soft tissue changes must also be considered, as posterior movement of the upper incisors affects lip support, facial profile balance, and gingival contour stability.

Therefore, in light of the need to assess the side effects associated with any adjunctive procedure in orthodontic treatment before recommending it for clinical use. This study aimed to evaluate external root resorption and periodontal indices during en‐masse retraction of maxillary anterior teeth stimulated with low‐intensity electrical currents. The null hypotheses of this trial were that there were no differences between the two upper anterior teeth en‐masse retraction groups in terms of the external root resorption and periodontal indices.

## 2 Methods and Materials

### 2.1 Trial Registration and Settings

This two‐parallel arms RCT was registered in the Clinical Trials database under the registration number (NCT06873490). Also, to ensure the ethical aspects of the research, the ethical approval for this trial protocol was obtained from the local Scientific Research Ethics Committee at Damascus University before starting the clinical phase (DN‐040423‐39). This randomized trial was reported in accordance with the CONSORT 2025 Statement (Hopewell et al. 2025).

All clinical procedures related to the orthodontic treatment of patients were performed in the Orthodontics Department at Damascus University Faculty of Dentistry. This study was funded by the University of Damascus (Ref. No.: 501100020595).

### 2.2 Estimate of Sample Size

The sample size was estimated using the Minitab Version 17 program (Minitab Inc., State College, Pennsylvania, the United States). A total of 30 patients were required in this trial based on the following assumptions: the least clinically important difference was 0.5 mm of root resorption, and the standard deviation of this variable was 0.47 mm (Kocadereli et al. 2011). The significance level was 0.05, the power of the study was 80%, and the statistical test used was a two‐samples *t*‐test. Finally, 34 patients were needed, after adding two patients to each group to consider any withdrawals.

### 2.3 Participants, the Inclusion and Exclusion Criteria

Young adult patients who visited the Department of Orthodontics at Damascus University—Faculty of Dentistry between November 2023 and March 2024 were examined. Patients registered in the archive records were also recalled if the initial diagnosis of their case met the inclusion criteria.

Patients were included in the study sample after being carefully examined by the researcher to ensure that they met the following inclusion criteria: Adult patients (18–25 years) with Class II division I malocclusion, good oral health and healthy periodontal tissues, camouflage treatment by extracting the upper first premolars and en‐masse retraction of the upper anterior teeth, the overjet 5–10 mm, the overbite 0%–33%, the presence of all upper and lower permanent teeth, not including the third molars, and good overall health. Exclusion criteria included patients with previous trauma to the front teeth, any health condition or long‐term medication affecting orthodontic treatment, active periodontal diseases, or contraindications for electrotherapy, such as pregnancy, epilepsy, or the presence of a cardiac pacemaker (Kasat et al. 2014). After providing a comprehensive explanation of the trial details and addressing all questions, informed consent was obtained from all patients who met the inclusion criteria and agreed to participate in the study.

### 2.4 Random Distribution, Blinding, and Allocation Concealment

Patients accepted into the study were randomly assigned to one of two groups. Random allocation was performed using the block randomization method. The block size was 8 patients, maintaining a 1:1 allocation ratio. The first group was the CER group, and the second group was the ESER group. Randomization and allocation sequence concealment were performed by one of the academic staff members in the Department of Orthodontics, Faculty of Dentistry, Damascus University. Randomization was achieved using random numbers generated by Minitab version 17 (Minitab Inc., State College, Pennsylvania, the United States). To ensure allocation sequence concealment, sealed and opaque envelopes with serial numbers were used.

Due to the nature of the current study, where the electrical stimulation device was applied only to one group, blinding was not feasible for the patients receiving treatment or the orthodontist administering it. However, blinding measures were implemented during data collection and analysis to minimize potential biases. Specifically, all panoramic radiographs and periodontal assessment forms were anonymized and assigned coded identifiers. The evaluators responsible for data extraction and statistical analysis were blinded to group allocation, ensuring an objective assessment of outcomes.

### 2.5 Treatment Sequence

#### 2.5.1 The First Stage: Leveling and Alignment of the Upper Arch

Before starting the leveling and alignment procedures on the maxillary dental arch, skeletal anchorage was secured on the maxillary dental arch to prevent the mesial slipping of the upper posterior teeth using self‐drilling mini‐implants (1.6 × 8 mm; 3S screw, Hubit, Seoul, Korea) that were applied between the roots of the maxillary first molars and the second premolars. A periapical radiograph of the root area of the second premolars and the maxillary first molars was performed, before and after the insertion of the implants, to confirm the integrity of the mini‐implant relationship with the adjacent structures. After that, they were bonded to these teeth using metal ligature wires. The patients were referred for the extraction of the maxillary first premolars at the Department of Maxillofacial Surgery, Damascus University, Faculty of Dentistry. The same resident surgeon extracted all the premolars in the sample.

Fixed orthodontic appliances with MBT prescription and 0.022‐inch bracket slot (Votion, Ortho Technology, Florida, the United States) were used in this study. A uniform wire sequence was followed in both groups, starting from 0.014‐inch NiTi wire until the 0.019 × 0.025‐inch stainless steel basic archwire was reached, with a 3‐week interval between each two wires. The sequence of the archwires used was as follows: 0.014‐inch NiTi, 0.016‐inch NiTi, 0.016 × 0.022‐inch NiTi, 0.017 × 0.025‐inch NiTi, 0.019 × 0.025‐inch NiTi, and 0.019 × 0.025‐inch SS. The last SS archwire was left for 3 weeks and then was replaced with a wire of the same type with 8 mm height crimpable hooks distal to the canines.

#### 2.5.2 Second Stage: The Upper Anterior Teeth En‐Masse Retraction

**Figure 1:**
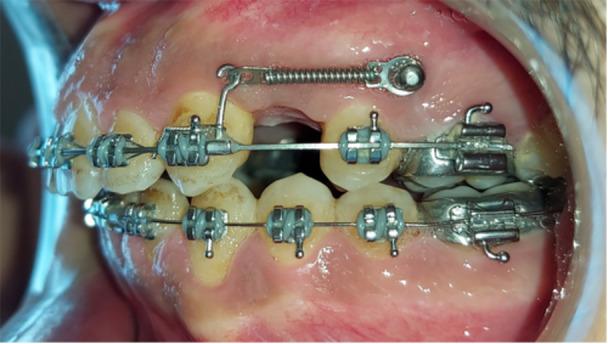
The conventional mini‐implants anchored en‐masse retraction of upper anterior teeth.

The sliding technique was used for en‐masse retraction in both study groups. An orthodontic force of approximately 500 g (250 g on each side) was applied to the crimpable hooks using bilateral closed‐coil nickel titanium springs (NT3 closed‐coil springs, American Orthodontics, Sheboygan, Wisconsin, the United States) anchored to orthodontic mini‐screws (Figure 1). Patients were followed up every 2 weeks during this phase to adjust the springs as needed to maintain a constant force (Khlef et al. 2020). The endpoint of en‐masse retraction in both groups was determined when the maxillary canines achieved a Class I relationship with the mandible and a normal incisor relationship.

#### 2.5.3 The Application of Low‐Intensity Electrical Stimulation in the ESR Group

**Figure 2:**
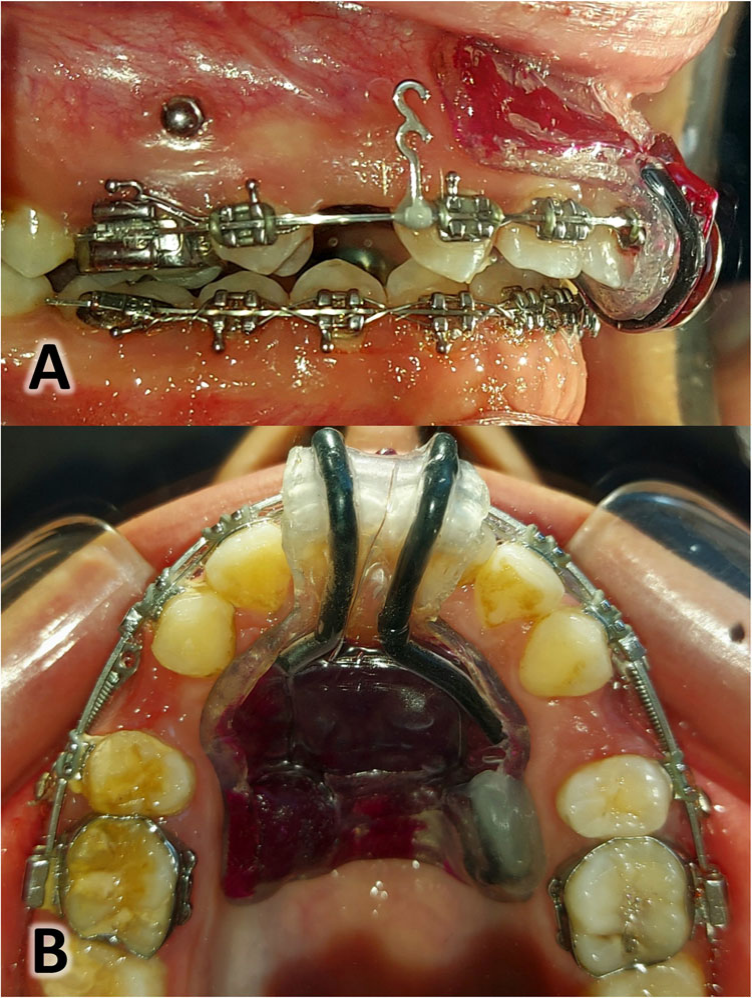
The electrical stimulation device. A: Lateral view of the dental arch with the device placed. B: Occlusal view of the device.

Patients in the electrical stimulation group were asked to use a specially fabricated removable device to apply the required electrical stimulation with a specific intensity to the anterior teeth. A custom‐made removable device was designed for each patient based on the design proposed by Shaadouh et al. in a previous study (Figure 2) (Shaadouh et al. 2023). The electrical current was applied according to the protocol proposed by Shaadouh et al. for continuous 5 h daily and during the whole en‐masse retraction phase. Patients were asked to inform the researcher immediately in case of any undesirable effects resulting from the device, such as ulceration and others, to take the appropriate procedures immediately or stop the treatment. The importance of full commitment to wearing the device and the necessity of reporting in case of poor adherence to the device were emphasized. Participants were allowed to withdraw voluntarily at any stage of the study without consequences. Additionally, exclusion occurred under the following circumstances: Noncompliance with treatment protocols, including inconsistent use of the electrical stimulation device. Development of adverse effects, such as excessive discomfort, periodontal complications, or severe ulceration. Orthodontic appliance failure, requiring adjustments outside the planned study protocol.

### 2.6 Outcomes Measure

#### 2.6.1 Root Resorption Assessment

To evaluate EARR, digital panoramic radiographs were taken at three time points: before orthodontic treatment (T0), before en‐masse retraction (T1), and after en‐masse retraction (T2). The ImageJ software (NIH and LOCI, Madison, Wisconsin, the United States) was used to analyze these radiographs. A straight line was drawn between the mesial and distal points of the cementoenamel junction (CEJ). Vertical lines were then drawn from the root apex and midincisal edge to the CEJ line to measure root and crown lengths.

The constant crown length between successive images was used as a reference to account for potential magnification differences between radiographs. This method was originally described by Linge and Linge (Linge and Linge 1983) and used in other studies (Gay et al. 2017; Khlef et al. 2020). The amount of root resorption (in mm) was calculated using the following equation: Root Resorption = Root Length (T0) − (Root Length (T1) × Correction Factor). The Correction Factor was calculated by dividing the Crown Length (T0) by the Crown Length (T1).

#### 2.6.2 Periodontal Status Assessment

The following periodontal indices were carefully evaluated by the researcher (R.I.S.): The GI, the papillary bleeding index (PBI), the dental plaque index (DPI), and the gingival recession index (GRI). The assessments were conducted on the upper anterior teeth at three distinct time points: before the fixed appliance was applied (T0), before en‐masse retraction initiation (T1), and at the end of the en‐masse retraction phase (T2).

The GI was assessed using a William's probe and a mouth mirror to evaluate the gingivitis severity in the following areas: the mesiobuccal papilla, the distobuccal papilla, the buccal gingival margin, and the lingual gingival margin (Silness and Löe 1964). The index scores were recorded as follows: Grade 0: Normal gingiva, Grade 1: Mild inflammation, slight discoloration, slight swelling, no bleeding on probing; Grade 2: Moderate inflammation, redness, edema, smooth glossy surface, bleeding on probing; and Grade 3: Severe inflammation, marked redness and swelling, ulceration, tendency to spontaneous bleeding. The GI value for each patient was determined by calculating the mean of the values of the six upper teeth.

The PBI was assessed using William's probe and a mouth mirror to evaluate the bleeding severity on the papillae's mesial and distal aspects (Mühlemann 1977). The index scores were recorded as follows: Grade 0: No bleeding is seen; Grade 1: One bleeding spot after probing the mesial and distal gingival sulcus; Grade 2: A small line of bleeding or several bleeding spots; Grade 3: The interdental space is filled with blood; and Grade 4: Profuse bleeding immediately after probing fills the interdental space and covers part of the gingiva or tooth. The PBI value for each patient was determined by calculating the mean of the values of the six upper teeth.

The DPI was assessed using a dental explorer and a mouth mirror, after air‐drying the teeth surfaces, to evaluate the thickness of the plaque at the following surfaces: buccal, lingual, mesiobuccal, and distobuccal surfaces. The index scores were recorded as follows: Grade 0: No plaque; Grade 1: A very thin layer of plaque on the gingival margin, visible only when scraped with a probe; Grade 2: A moderate amount of plaque along the gingival margin, visible to the naked eye, with no plaque in the interdental spaces; and Grade 3: Severe plaque accumulation along the gingival margin and in the interdental spaces. The DPI value for each patient was determined by calculating the mean of the values of the six upper teeth (Al‐Ibrahim et al. 2022).

The presence of gingival recession on the studied teeth was determined using a William's probe, visual inspection, and direct clinical measurement from the CEJ to the free gingival margin if recession was present.

#### 2.6.3 Statistical Analysis

The SPSS software (version 25.0; IBM, Armonk, New York, the United States) was used to conduct all statistical tests. The Shapiro–Wilk test was utilized to evaluate the normality of data distribution. To compare the amount of external root resorption between the two groups, the Mann–Whitney *U* test was applied. The *χ* ^2^ test was used to analyze the number of teeth affected by resorption after the leveling and alignment stage, as well as the en‐masse retraction stage, across the two groups. The independent‐samples *t*‐test or Mann–Whitney *U* test was employed to identify significant differences in periodontal indices between the groups. Additionally, the Friedman test or one‐way repeated measures ANOVA was applied to assess significant changes in periodontal variables over time within each group.

#### 2.6.4 Reliability Assessment of the Method Used

A single examiner performed all radiographic measurements to ensure consistency. Root lengths of the anterior teeth were measured on the panoramic image in the same manner as previously described. Assessments were performed twice, with a 2‐week interval between each assessment. The intraclass correlation coefficients (ICCs) were used to determine any random errors, and paired‐samples *t*‐tests were used to detect systematic errors.

## 3 Results

### 3.1 Patient Recruitment and Baseline Characteristics of the Sample

**Figure 3:**
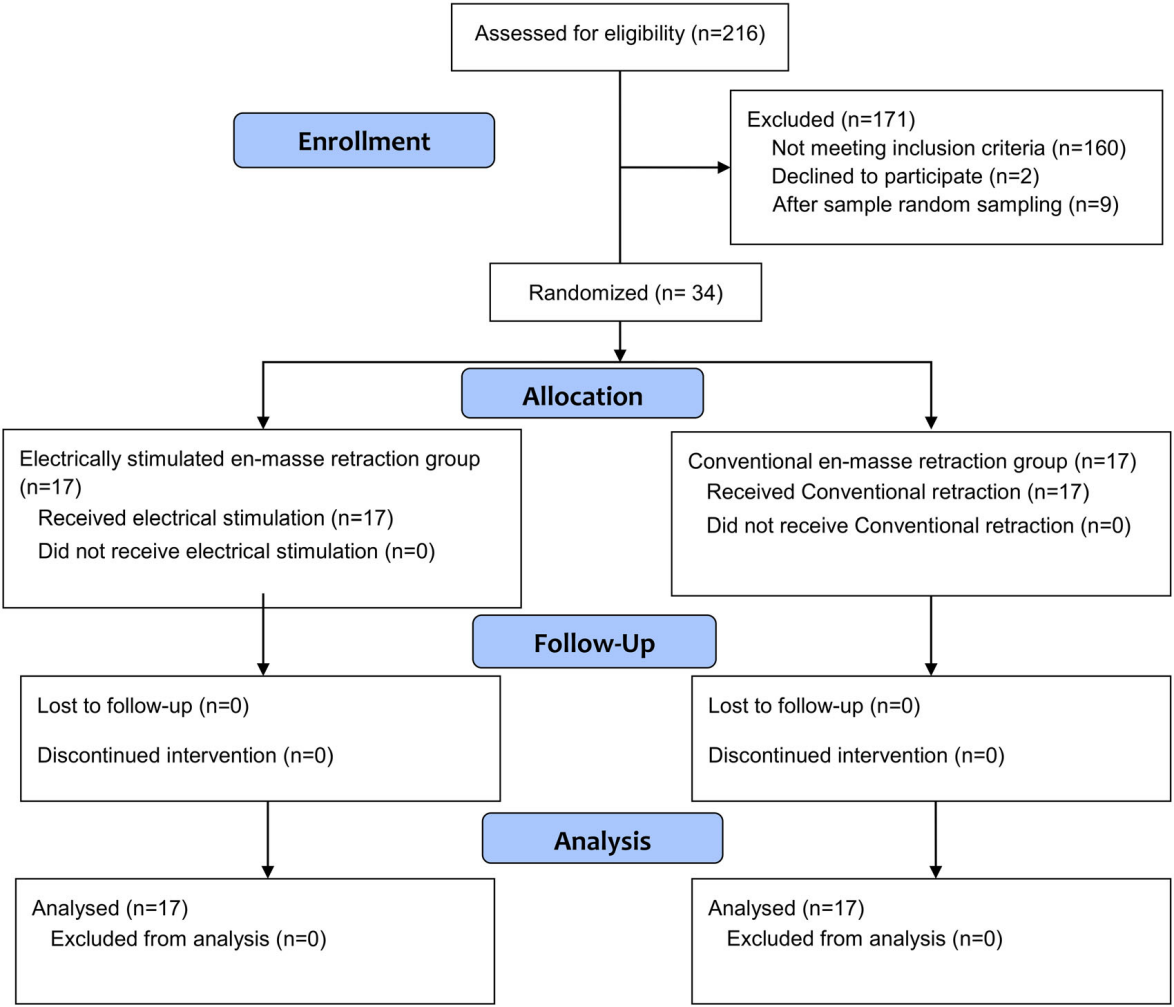
CONSORT flow diagram of patient recruitment, follow‐up, and entry into data analysis.

A total of 216 patients were initially screened for inclusion in this RCT by the researcher (R.I.S.) under the supervision of the coauthor (M.Y.H.). After applying the inclusion criteria, 171 patients were excluded, 2 declined participation, and 9 were excluded through simple random sampling. The final study cohort comprised 34 patients: 26 females (76.5%) and 8 males (23.5%) with a mean age of 21.12 ± 2.41 years. Seventeen patients were randomly assigned to each treatment group. Baseline comparisons revealed no statistically significant differences between groups regarding age (*p* = 0.567) or sex (*p* = 0.419). Table 1 summarizes the baseline characteristics of the study sample. All participants in both groups successfully completed the treatment and all scheduled follow‐up appointments, with no dropouts. Figure 3 shows the CONSORT flow diagram of patients' recruitment, follow‐up, and entry into data analysis.

**Table 1:** Baseline characteristics of the sample at the beginning of the treatment.

Variable		ESER group		CER group		Total		*p* value
		*n*	%	*n*	%	*n*	%	
Gender	Male	3	17.6	5	29.4	8	23.5	0.419^a^
	Female	14	82.4	12	70.6	26	76.5	
Age	Mean ± SD	20.88 ± 2.29		21.36 ± 2.56		21.12 ± 2.41		0.567^b^

Abbreviations: CER: conventional en‐masse retraction, ESER: electrically stimulated en‐masse retraction, SD: standard deviation.

^a^ *χ* ^2^ test.

^b^ Independent‐samples *t*‐test.

### 3.2 Measurement of Reliability

The intraclass correlation coefficients (ICCs) ranged from 0.993 to 0.999, indicating excellent intra‐examiner reliability for all measurements (Table S1). Paired‐samples *t*‐tests revealed no statistically significant differences between repeated measurements, confirming the absence of systematic measurement errors (Table S1).

### 3.3 Root Resorption Assessment

In both groups, minimal root resorption was observed in the leveling and alignment and en‐masse retraction phase. The external root resorption following the leveling and alignment phase ranged between 0.36 and 0.95 mm in the ESER group and 0.49 and 0.93 mm in the CER group, with no statistically significant difference between groups (*p* > 0.05; Table 2). Furthermore, no significant difference in root resorption was observed at the end of the en‐masse retraction phase between the two groups (*p* > 0.05; Table 2). The observed root resorption in the ESER and CER ranged between 0.27 and 0.64 and 0.32 and 0.71 mm, respectively. Maxillary lateral incisors exhibited the greatest root resorption, followed by central incisors, in both groups (Table 2).

**Table 2:** Root resorption measurements during leveling, alignment, and en‐masse retraction phases in both groups.

Phase	Tooth	ESER group (*n* = 17)					CER group (*n* = 17)						95% Confidence interval of the difference	*p* value^a^
		Mean	Median	SD	Min	Max	Mean	Median	SD	Min	Max	Mean difference		
T0 –T1	13	0.36	0	0.53	0	1.51	0.49	0	0.63	0	1.56	−0.13	−0.54, 0.28	0.582
	12	0.95	0.7	1.06	0	3.69	0.93	0.7	1.00	0	2.85	0.01	−0.71, 0.73	0.986
	11	0.79	0.5	0.93	0	2.8	0.71	0.5	0.87	0	2.54	0.08	−0.55, 0.71	0.747
	21	0.72	0.5	0.85	0	2.55	0.78	0.5	0.94	0	2.99	−0.06	−0.69, 0.57	0.943
	22	0.88	0.5	0.91	0	2.34	0.85	0.5	1.00	0	2.6	0.03	−0.64, 0.69	0.789
	23	0.43	0	0.64	0	1.82	0.51	0	0.66	0	1.76	−0.08	−0.54, 0.37	0.762
T1 –T2	13	0.27	0	0.59	0	2.15	0.32	0	0.69	0	2.53	−0.04	−0.49, 0.41	0.756
	12	0.64	0	0.97	0	3.1	0.71	0	0.92	0	2.9	−0.06	−0.73, 0.6	0.820
	11	0.50	0	0.72	0	229	0.65	0	0.95	0	2.88	−0.15	−0.74, 0.44	0.791
	21	0.58	0	0.86	0	2.49	0.77	0	1.07	0	2.84	−0.19	−0.87, 0.48	0.636
	22	0.68	0	1.01	0	2.87	0.74	0	1.09	0	2.98	−0.06	−0.79, 0.67	0.719
	23	0.32	0	0.64	0	2.42	0.41	0	0.88	0	3.18	−0.09	−0.63, 0.45	0.983

Abbreviations: 13: the upper right canine, 12: the upper right lateral incisor, 11: the upper right central incisor, 21: the upper left central incisor, 22: the upper left lateral incisor, 23: the upper left canine, CER: conventional en‐masse retraction, ESER: electrically stimulated en‐masse retraction, SD: standard deviation, T0: before orthodontic treatment, T1: before en‐masse retraction, T2: after en‐masse retraction, T0–T1: leveling and alignment phase, T1–T2: en‐masse retraction phase.

^a^ Mann–Whitney *U* test.

During the leveling and alignment stage, 56.6% of teeth exhibited root resorption in the electrical stimulation group, compared to 52% in the conventional retraction group. This percentage difference was not statistically significant (*p* = 0.482; Table 3). Following the en‐masse retraction stage, the percentage of teeth exhibiting root resorption significantly decreased in both groups (*p* < 0.001, *p* = 0.027, respectively; Table 4), reaching 36.3% and 41.2%, respectively. However, there was no significant difference between the groups in this stage either (*p* = 0.472; Table 3).

**Table 3:** Number of teeth affected by resorption following the leveling, alignment, and en‐masse retraction stages.

Phase	Variable	ESER group (*n* = 17)		CER group (*n* = 17)		*p* value^a^
		*n*	%	*n*	%	
T0–T1	No resorption	44	43.1%	49	48.0%	0.482
	Resorption	58	56.9%	53	52.0%	
T1–T2	No resorption	65	63.7%	60	58.8%	0.472
	Resorption	37	36.3%	42	41.2%	

Abbreviations: CER: conventional en‐masse retraction, ESER: electrically stimulated en‐masse retraction, SD: standard deviation, T0: before orthodontic treatment, T1: before en‐masse retraction, T2: after en‐masse retraction, T0–T1: leveling and alignment phase, T1–T2: en‐masse retraction phase.

^a^ *χ* ^2^ test.

**Table 4:** Number of teeth affected by resorption during en‐masse retraction and changes over time in each group.

Variable		ESER group (*n* = 17)			CER group (*n* = 17)		
		T1–T2			T1–T2		
		No resorption	Resorption	*p* value^a^	No resorption	Resorption	*p* value^a^
T0–T1	No resorption	42	2	< 0.001*	44	5	0.027*
	Resorption	23	35		16	37	

Abbreviations: CER: conventional en‐masse retraction, ESER: electrically stimulated en‐masse retraction, T0: before orthodontic treatment, T1: before en‐masse retraction, T2: after en‐masse retraction, T0–T1: leveling and alignment phase, T1–T2: en‐masse retraction phase.

^a^ McNemar test.

* Significant at the 0.05 level.

### 3.4 Periodontal Status Assessment

Mild gingivitis was observed in both groups at T0, T1, and T2 (0.26, 0.50, and 0.44, respectively, in the ESER group, and 0.29, 0.46, and 0.54, respectively, in the CER group; Table 5). Furthermore, no statistically significant differences in gingival inflammation were found between the two groups at any evaluation time point (Table 5). On the other hand, in both groups, the GI exhibited a significant increase at the end of both the leveling and alignment phase and the en‐masse retraction phase compared to baseline values (0.24 and 0.17 in ESER group and 0.17 and 0.25 in CER group, respectively; Tables 6 and 7).

**Table 5:** Periodontal indices before orthodontic treatment, before en‐masse retraction, and after en‐masse retraction in both groups.

Variable	Time	ESER group (*n* = 17)					CER group (*n* = 17)					Mean difference	95% Confidence interval of the difference	*p* value
		Mean	Median	SD	Min	Max	Mean	Median	SD	Min	Max			
GI	T0	0.26	0.27	0.17	0	0.6	0.29	0.31	0.16	0	0.48	−0.03	−0.15, 0.08	0.568^a^
	T1	0.50	0.50	0.15	0.25	0.9	0.46	0.48	0.19	0.1	0.83	0.04	−0.08, 0.16	0.499^a^
	T2	0.44	0.38	0.16	0.2	0.73	0.54	0.55	0.18	0.25	0.88	−0.09	−0.21, 0.03	0.131^a^
PBI	T0	0.20	0.17	0.15	0	0.46	0.23	0.21	0.18	0	0.63	−0.03	−0.15, 0.08	0.581^a^
	T1	0.34	0.33	0.18	0	0.75	0.29	0.30	0.15	0	0.73	0.04	−0.07, 0.16	0.475^a^
	T2	0.28	0.28	0.17	0	0.63	0.33	0.33	0.14	0.1	0.55	−0.05	−0.15, 0.06	0.387^a^
DPI	T0	0.33	0.33	0.21	0	0.63	0.33	0.35	0.21	0.02	0.67	0.01	−0.14, 0.15	0.931^a^
	T1	0.54	0.53	0.18	0.33	0.9	0.53	0.50	0.19	0.23	1	0.01	−0.12, 0.13	0.926^a^
	T2	0.64	0.53	0.35	0.25	1.5	0.64	0.53	0.30	0.28	1.25	0.00	−0.22, 0.23	0.890^b^
GRI	T0	0.00	0.00	0.00	0	0	0.00	0.00	0.00	0	0	0.00		1.00^b^
	T1	0.00	0.00	0.00	0	0	0.00	0.00	0.00	0	0	0.00		1.00^b^
	T2	0.00	0.00	0.00	0	0	0.00	0.00	0.00	0	0	0.00		1.00^b^

Abbreviations: CER: conventional en‐masse retraction, DPI: dental plaque index, ESER: electrically stimulated en‐masse retraction, GI: gingival index, GRI: gingival recession index, PBI: papillary bleeding index, T0: before orthodontic treatment, T1: before en‐masse retraction, T2: after en‐masse retraction.

^a^ Independent‐samples *t*‐test.

^b^ Mann–Whitney *U* test.

**Table 6:** Periodontal indices before orthodontic treatment, before en‐masse retraction, and after en‐masse retraction with changes over time in each group.

Variable	Time	ESER group (*n* = 17)			*p* value	CER group (*n* = 17)			*p* value
		Mean	Median	Std. deviation		Mean	Median	Std. deviation	
GI	T0	0.26	0.27	0.17	< 0.001^a^ ^,^ *	0.29	0.31	0.16	< 0.001^a^^,^ *
	T1	0.50	0.50	0.15		0.46	0.48	0.19	
	T2	0.44	0.38	0.16		0.54	0.55	0.18	
PBI	T0	0.20	0.17	0.15	0.013^a^^,^ *	0.23	0.21	0.18	0.203^a^
	T1	0.34	0.33	0.18		0.29	0.30	0.15	
	T2	0.28	0.28	0.17		0.33	0.33	0.14	
DPI	T0	0.33	0.33	0.21	0.010^b^ ^,^ *	0.33	0.35	0.21	0.028^b^ ^,^ *
	T1	0.54	0.53	0.18		0.53	0.50	0.19	
	T2	0.64	0.53	0.35		0.64	0.53	0.30	
GRI	T0	0.00	0.00	0.00	1.00^b^	0.00	0.00	0.00	1.00^b^
	T1	0.00	0.00	0.00		0.00	0.00	0.00	
	T2	0.00	0.00	0.00		0.00	0.00	0.00	

Abbreviations: CER: conventional en‐masse retraction, DPI: dental plaque index, ESER: electrically stimulated en‐masse retraction, GI: gingival index, GRI: gingival recession index, PBI: papillary bleeding index, T0: before orthodontic treatment, T1: before en‐masse retraction, T2: after en‐masse retraction.

^a^ One‐way repeated measures ANOVA.

^b^ Friedman test.

* Significant at the 0.05 level.

**Table 7:** Mean differences in periodontal indices between each study time point.

Variable	Phase	ESER group (*n* = 17)		CER group (*n* = 17)	
		Mean difference	*p* value^a^	Mean difference	*p* value^a^
GI	T0 VS T1	−0.24	< 0.001*	−0.17	0.010*
	T0 VS T2	−0.17	0.001*	−0.25	< 0.001*
	T1 VS T2	0.06	0.412	−0.08	0.681
PBI	T0 VS T1	−0.14	0.043*		
	T0 VS T2	−0.09	0.142		
	T1 VS T2	0.05	0.663		
DPI	T0 VS T1	−0.20	0.077	−0.20	0.077
	T0 VS T2	−0.31	0.011*	−0.31	0.049*
	T1 VS T2	−0.11	1.00	−0.11	1.00

Abbreviations: CER: conventional en‐masse retraction, DPI: dental plaque index, ESER: electrically stimulated en‐masse retraction, GI: gingival index, GRI: gingival recession index, PBI: papillary bleeding index, T0: before orthodontic treatment, T1: before en‐masse retraction, T2: after en‐masse retraction.

^a^ Bonferroni test.

* Statistically significant difference at *p* < 0.05.

PBI values were greater in the CER group at the end of the retraction phase (T2) compared to the low‐intensity electrical stimulation group (0.33 and 0.28, respectively; Table 5). No significant differences were observed between both groups at the initial assessment (0.20 and 0.23, respectively; *p* = 0.581), at the end of the leveling and alignment phase (0.34 and 0.29, respectively; *p* = 0.475), and after the en‐masse retraction phase (0.28 and 0.33, respectively; *p* = 0.387).

Plaque index values were small at all assessment times (T0, T1, and T2) in both groups and indicate good oral health (0.33, 0.54, and 0.64, respectively, in the ESER group, and 0.33, 0.53, and 0.64, respectively, in the CER group; Table 5). Mean plaque index values were almost equal between the two en‐masse retraction groups at all time points. No significant differences were observed between the two groups at T0, T1, and T3 (*p* = 0.931, *p* = 0.926, and *p* = 0.890 respectively; Table 5). In both groups, the Plaque index exhibited a significant increase at the end of the en‐masse retraction phase compared to baseline values (0.31 and 0.31 in ESER and CER, respectively; Tables 6 and 7). No gingival recession was observed on any of the teeth examined in all subjects in both groups (Table 5).

### 3.5 Harms

No adverse effects or side effects were observed during the trial.

## 4 Discussion

Among the most common complications of fixed orthodontic treatment, root resorption and periodontitis may pose challenges for orthodontists (Ahmed et al. 2022; Yassir et al. 2021). Many researchers have sought to find suitable means to mitigate these complications during treatment. The present study aimed to evaluate these complications when low‐intensity electrical stimulation was combined with orthodontic treatment, especially in cases of upper incisors en‐masse retraction.

The follow‐up interval during en‐masse retraction in this study was set at 2 weeks. This decision was based on the need for closer monitoring of treatment progression. More frequent evaluations allowed for early identification of biological responses, ensuring precise adjustments to treatment protocols as needed. Additionally, shorter intervals facilitated better control over patient compliance with the electrical stimulation device, which is crucial for achieving optimal results. Although traditional orthodontic treatment commonly employs longer intervals, many previous studies used 2‐week interval during the en‐masse retraction phase (Khlef et al. 2020; Mousa et al. 2023; Shaadouh et al. 2024a, 2024b).

In this study, digital panoramic radiography was selected to assess root resorption during en‐masse retraction, with images taken at three time points: before treatment initiation (T0), before en‐masse retraction (T1), and after retraction completion (T2). Although periapical radiographs provide greater resolution for localized assessments, panoramic imaging was preferred due to its ability to capture a broader view of all upper anterior teeth in a single exposure, minimizing radiation exposure for the patient. Given the study's need to evaluate multiple teeth simultaneously, periapical radiographs would have required multiple exposures, increasing cumulative radiation dosage. To enhance measurement accuracy, all panoramic images were digitally calibrated, and evaluations were performed using standardized radiographic analysis protocols. Many previous studies have used these techniques to evaluate external root resorption (Futyma‐Gąbka et al. 2022; Huang et al. 2010; Khlef et al. 2020; Kurnaz and Buyukcavus 2024)

A slight root resorption after the completion of the leveling and alignment phase was observed in both groups of retraction, which may be classified within the limits of minimal root resorption (less than 10% of the root length), as reported by Inchingolo et al. (2024). Therefore, this root resorption was not clinically significant. This may be attributed to the sequential gradation of the orthodontic wires, which contributed to generating low and biologically acceptable continuous force levels, which translates approximately to intermittent force due to the elastic nature of the periodontal ligament (Cattaneo et al. 2009).

Also, at the end of the en‐masse retraction phase, external root resorption was found on many of the examined teeth in both groups. Although the root resorption was smaller and accrued in fewer teeth in the ESER group compared to the CER group (36.3% and 41.2%, respectively), there was no statistically significant difference between the two retraction groups in the average amount of root resorption obtained on each of the studied teeth. This result suggests that low‐intensity electrical stimulation may not impact the extent of root resorption. However, it is important to note that the teeth affected by the root absorption in the en‐masse retraction phase had already been affected by root resorption in the leveling and alignment stage.

The incidence of root resorption in this study may be explained by the fact that the risk of root resorption is increased in cases of en‐masse retraction of the six maxillary anterior teeth due to the large amount of retraction movement usually required in these cases (Liou and Chang 2010).

After reviewing the existing literature, no previous studies were found that evaluated the effect of applying low‐intensity electrical stimulation on the amount of root resorption, with the exception of one pilot study, which evaluated the effect of electrical stimulation on root resorption during en‐masse retraction (Shaadouh et al. 2023). This preliminary study showed a mild external root resorption in 30% of the evaluated teeth. That result was similar to the current study.

Concerning other controlled clinical studies that have evaluated root absorption in cases of en‐masse retraction, whether traditional or stimulated by various means. The results of the current study agreed with those of Khlef et al. who reported the occurrence of mild apical root resorption during the corticotomy‐assisted en‐masse retraction phase. The root resorption in their study ranged between 0% and 6% (Khlef et al. 2020), which was almost similar to this study. In contrast, the results of the current study differed from those of Yousry et al. (Yousry et al. 2015). They evaluated the effect of low‐level laser on the amount of root resorption in cases of mini‐implant‐supported en‐masse retraction of the upper six anterior teeth. Their study showed that root resorption had occurred in the roots of all the studied teeth. The average root resorption was 1.16 mm in the low‐level laser group compared to 1.31 mm in the control group, with no significant difference between the two groups (Yousry et al. 2015). The disagreement may be attributed to the difference in the method of studying and measuring root resorption between the two studies. The high resolution of the CBCT image used in their study allows the detection of small amounts of root resorption that cannot appear on the periapical or panoramic radiograph, thus leading to more accurate evaluation and results. Huang et al. reported a root resorption during CER of approximately 0.43 and 0.58 mm in the central and lateral incisors, respectively. This was almost similar to the present study (0.50 and 0.64, respectively, in the low‐intensity electric stimulation group, 0.65 and 0.71, respectively, in the conventional retraction group). However, it should be noted that the amount of applied retraction force was different between the two studies, as a force of 250 g was applied to each side in the present study, while Huang et al. used a force of 150 g to each side to achieve the en‐masse retraction (Huang et al. 2010).

Four periodontal indices were used to assess the periodontal tissue condition in this study, which was supposed to give a clear picture of the periodontal condition. Considering that this study is the first controlled clinical study that evaluated the effect of low‐intensity electrical stimulation during orthodontic tooth movement on the periodontal condition, the results of the present study showed that the values of all measured periodontal parameters were almost equal between the ESER and CER groups before starting orthodontic treatment (T0). All of these values were classified as mild gingivitis. This may be attributed to the fact that patients who were suitable for the study according to the inclusion criteria were directed to undergo periodontal treatment and adhere to oral health instructions before being included in the study sample (Hussain et al. 2024).

A slight increase was recorded in the GI, PBI, and plaque index after the completion of leveling and alignment and before starting the en‐masse retraction (T1) in both groups. No significant differences were found between the two groups. The reason for this increase may be attributed to the increased accumulation of dental plaque due to the presence of fixed orthodontic appliances and their components, which increases the possibility of gingivitis. This result was consistent with most previous studies in this field (Al‐Ibrahim et al. 2022; Khlef and Hajeer 2022; Pandis et al. 2007). This increase in the values of the gingival indices during the leveling and alignment stage was statistically but not clinically significant, as these indices remained within the limits of a simple degree. This may be attributed to the strict and frequent oral hygiene instructions given to the patients, in addition to the regular patient visits during the follow‐up period (once every 3 weeks), which in turn kept the plaque index values within the acceptable and non‐destructive limits for the periodontal tissues. No gingival recession was recorded in any of the teeth in both groups due to the absence of predisposing factors, such as severe plaque accumulation and severe gingivitis (Melsen and Allais 2005), in conjunction with the application of controlled forces during alignment and leveling.

At the final evaluation time (after completion of the en‐masse retraction), although the plaque index was similar in the ESER and CER groups, the GI and PBI values were smaller in the electrical stimulation group (0.44 and 0.28, respectively) compared to the conventional retraction group (0.54 and 0.33, respectively). However, no significant differences between the two groups in terms of the GI, PBI, and plaque index were found. This suggests that there was no significant effect of electrical stimulation on the gingival status during orthodontic treatment.

This increase in the values of the periodontal indices was statistically but not clinically significant, as these indices remained within the simple grade. The increase in these values in both groups may be attributed to the presence of the fixed orthodontic appliance and the added retraction springs and hooks distal to canines. It made ideal tooth brushing more difficult for the patients, which contributed to the accumulation of a greater amount of plaque and the accompanying inflammation and bleeding of the gingiva (Khlef and Hajeer 2022).

The reason why gingival recession did not occur with either retraction technique may be that plaque levels and applied orthodontic forces remained within acceptable, non‐destructive levels of the periodontal tissues.

After reviewing the literature, no previous study was found that evaluated the effect of electrical stimulation on periodontal indices either with the en‐masse retraction of the upper anterior six teeth or other orthodontic tooth movements, except for the study by Lee et al. which evaluated the effect of electrical stimulation on teeth and periodontal tissues using a special electric toothbrush (Proxywave, Tromatz basic, proxyhealthcare, Ulsan, Korea) during orthodontic treatment (Lee and Ha 2021). The results of the present study differed from those of Lee et al. They found that electrical stimulation using an electric toothbrush helped to significantly reduce the gingivitis index compared with a conventional electric toothbrush. They explained this result by the ability of electrical stimulation to affect the components of the accumulated bacterial plaque and lose its pathogenic properties (Lee and Ha 2021). This difference in the results may be attributed to the difference in the properties of the electrical current used, as the toothbrush used in Lee et al's study produced alternating and direct electrical currents together, while a low‐intensity direct electrical current was used in the present experiment. In addition, there was a difference in the method of applying electrical stimulation. In the current study, the electrical current was directed to the mucosal tissues around the upper anterior teeth, while in the study of Lee et al. it was applied to the dental and gingival tissues using an electric toothbrush.

Beyond electrical stimulation, nanoparticles, particularly those with antibacterial properties, present a promising avenue for improving periodontal health in orthodontic treatment. Studies have demonstrated their potential to reduce bacterial adhesion, enhance plaque control, and minimize gingival inflammation (Ahuja et al. 2025). Given their ability to mitigate microbial accumulation on orthodontic appliances, future research could explore integrating nanoparticle coatings with electrical stimulation technology to optimize oral hygiene and long‐term periodontal stability. Investigating the synergistic effects of these advancements may pave the way for more effective strategies to preserve periodontal health during orthodontic interventions.

### 4.1 Limitations of the Current Work

The effect of gender on root resorption or periodontal variables was not investigated in this study due to the small number of male patients in the sample. Therefore, it is necessary to investigate gender differences in future studies, especially since these variables may be affected by different hormonal changes between the sexes. Also, the experiment lasted for a relatively short period, only until the end of the en‐masse retraction. Therefore, the long‐term effects of electrical stimulation on root resorption and tooth vitality were not investigated. Finally, this technique requires optimal patient cooperation in wearing the electrical stimulation device to achieve the best results, and therefore, the results may be significantly affected if the patient does not adhere to the instructions. Therefore, it is necessary to investigate patient compliance in future studies or explore new techniques for applying electrical stimulation that do not depend on patient cooperation.

## 5 Conclusion

Based on the results of the present study, it could be concluded that low‐intensity electrical stimulation had no significant effect on root resorption during the en‐masse retraction of the six upper anterior teeth. Both groups of en‐masse retraction, whether stimulated by low‐intensity electrical current or conventional, exhibited comparable slight root resorption without any significant difference between the two groups. Additionally, low‐intensity electrical stimulation did not affect the periodontal status during en‐masse retraction, as no significant differences were observed between the two groups in periodontal variables.

## Author Contributions

Rashad I. Shaadouh recruited patients, performed the clinical procedures, collected the radiographs, measured the clinical outcomes, analyzed the collected data, interpreted the results, and wrote the first drafts of this paper. Mohammad Y. Hajeer supervised the whole research project, helped in data analysis and interpretation of the results, and corrected the early drafts of this manuscript. Mohammad Khursheed Alam co‐supervised this project, helped in statistical analysis, and participated in writing this manuscript. Samer T. Jaber participated in the inception of this work and study design, helped in data analysis and interpretation, and edited the final drafts of this manuscript. All authors read and approved the manuscript in its final form.

## Ethics Statement

This project was approved by the Biomedical Research Ethics Committee at the University of Damascus before starting data collection (DN‐040423‐39).

## Consent

The authors have nothing to report.

## Conflicts of Interest

The authors declare no conflicts of interest.

## Supporting information


**Supplementary Table 1:** Assessment of the systematic and random errors in the current study (n = 20).

## Data Availability

Datasets used and/or analyzed during the current study will be provided by the corresponding author upon reasonable request.
