# Supplementary material for: Effect of Low‐Intensity Electrical Stimulation on External Apical Root Resorption and Periodontal Indices Following En‐Masse Retraction of Upper Anterior Teeth in Young Adults: A Randomized Controlled Trial
Source: Clin Exp Dent Res. 2025 Aug 5;11(4):e70188. doi: 10.1002/cre2.70188 (PMC12323048; doi:10.1002/cre2.70188)
Supplement: Supplementary file 1 — Supplementary Table 1: Assessment of the systematic and random errors in the current study (n = 20). [file CRE2-11-e70188-s001.docx]

| **Supplementary Table 1: Assessment of the systematic and random errors in the performed measurements (n=20).** | | | | | | | |
| --- | --- | --- | --- | --- | --- | --- | --- |
| Variable | 1^ST^ measurement  Mean ± SD | 2^nd^ measurement  Mean ± SD | Mean Difference ± SD | Systematic error | Random error assessment | | |
|  |  |  |  | P-value^a^ | ICCs | 95% CI | |
|  |  |  |  |  |  | min | max |
| 11 | 15.37± 0.96 | 15.35± 0.95 | 0.02± 0.07 | 0.198 | 0.999 | 0.997 | 0.999 |
| 12 | 12.88± 1.55 | 12.92± 1.61 | -0.04± 0.12 | 0.133 | 0.997 | 0.992 | 0.999 |
| 13 | 18.6± 1.21 | 18.61± 1.23 | -0.01± 0.09 | 0.742 | 0.999 | 0.997 | 1.000 |
| 21 | 15.63± 0.95 | 15.63± 0.96 | 0.01± 0.08 | 0.617 | 0.997 | 0.992 | 0.999 |
| 22 | 13.11± 1.6 | 13.09± 1.59 | 0.02± 0.10 | 0.371 | 0.998 | 0.995 | 0.999 |
| 23 | `18.85± 1.34 | `18.86± 1.34 | -0.00± 0.04 | 0.652 | 1.000 | 0.999 | 1.000 |
| a: Paired-sample t-test, SD: standard deviation, Cl: Confidence Interval, 13: the upper right canine, 12: the upper right lateral incisor, 11: the upper right central incisor, 21: the upper left central incisor, 22: the upper left lateral incisor, 23: the upper left canine. | | | | | | | |
